# Efficacy of Autologous Micrografting Technology in Managing Osteoarthritis Pain: A Pilot Study

**DOI:** 10.3390/bioengineering11111119

**Published:** 2024-11-06

**Authors:** Camilo Partezani Helito, Valeria Pessei, Cecilia Zaniboni, Ilie Muntean

**Affiliations:** 1 Grupo de Joelho, Instituto de Ortopedia e Traumatologia, Hospital das Clínicas HCFMUSP, Faculdade de Medicina, Universidade de São Paulo, São Paulo 05403-010, Brazil; 2 Hospital Sírio Libanês, São Paulo 01308-901, Brazil; 3 Department Chemistry, Biology, Biotechnology, University of Perugia, 06123 Perugia, Italy; 4 SHRO Italia Foundation ETS, via Sestriere 17, 10060 Candiolo, Italy; 5 Fundació Hospital Sant Joan de Déu de Martorell, 08760 Barcelona, Spain

**Keywords:** osteoarthritis (OA), intra-articular injection, knee osteoarthritis, micrograft, pain management, regenerative medicine

## Abstract

Osteoarthritis (OA) is one of the most common joint diseases worldwide, predominantly present in elderly people. Being a major source of pain for patients, it is debilitating and leads inevitably to a reduction in quality of life. The management of OA needs a personalized and multidimensional approach, resulting in the emergence of new regenerative and non-invasive methods, such as the use of micrografts. In this pilot study, Rigenera^®^ Technology was employed to obtain micrografts of cartilage tissue to be injected into the knees of 10 patients with osteoarthritic pain. To assess the efficacy of the treatment concerning pain reduction at this site, patients were asked to complete KOOS and WOMAC questionnaire and a VAS test before and after the procedure. The results presented in this article show how Rigenera^®^ treatment can potentially improve OA symptoms, alleviating pain in patients.

## 1. Introduction

Osteoarthritis (OA) is a widespread condition that affects over 500 million people globally, making it one of the most common musculoskeletal disorders [1,2]. The prevalence of OA increases with age, with an estimated prevalence of 10% of men and 18% of women over 60 years of age [3,4]. Patients affected by OA show symptoms such as pain, stiffness, swelling, and limitations to motion in the affected area, thus reducing quality of life and having consequences on people’s mental health [5,6].

From a clinical point of view, OA is a degenerative inflammatory joint disease characterized by the progressive disintegration of cartilage, synovial fluid modifications, and bone erosion, leading to a change in the whole joint structure [7]. Based on etiology, it is possible to distinguish primary and secondary OA [8]. Primary OA, also known as idiopathic OA, is usually related to aging and genetic factors, while secondary OA is caused by an underlying condition or factor such as joint injuries or trauma, obesity, inflammatory diseases such as rheumatoid arthritis, and metabolic disorders [9,10].

The management of OA requires a multidimensional approach tailored to the individual patient’s needs and the severity of their condition. In the early stages, lifestyle changes such as weight reduction, regular and low-impact physical activity, and a more balanced diet rich in anti-inflammatory foods could improve the situation by increasing mobility and reducing pain [11,12]. However, in advanced cases, the standard procedures include pharmacological treatments such as analgesics, non-steroidal anti-inflammatory drugs (NSAIDs), intra-articular injections of corticosteroids, or hyaluronic acid [13,14]. Although these interventions provide short-term benefits, they can result in significant adverse complications for patients, including cardiovascular risks and gastrointestinal disorders [15]. Moreover, these treatments are not resolutive, often leading the physician to the only available solution for joint replacement, an artificial prosthesis [16,17]. In this context, researchers are investigating new therapeutic methods to reduce pain and improve patients’ quality of life through the employment of biological and regenerative therapies involving growth factors or cytokine inhibitors, platelet-rich plasma (PRP), and others. Specifically, in this study, a procedure based on the use of micrografts [18] will be investigated.

Rigenera^®^ Technology is an approach that can obtain autologous micrografts through the mechanical disaggregation of a small biopsy of the patient’s tissue, typically from the auricular cartilage [19,20,21]. Recently, the efficacy and safety of this procedure in patients with early-stage knee osteoarthritis was assessed by a study that showed significant improvements in knee stability, pain, swelling, mechanical locking, and overall mobility by using Rigenera^®^ Technology [22]. Overall, Rigenera^®^ technology offers a potentially effective approach to managing osteoarthritis, with encouraging preliminary results. The aim of the present study is to evaluate the effect of Rigenera^®^ treatment in the modulation of pain in patients with OA.

## 2. Materials and Methods

### 2.1. Clinical Study and Patients

This study was conducted from August 2022 to March 2023 and enrolled 10 patients (5 males and 5 females) with an average age of 55 years. Six patients were suffering from primary osteoarthritis, two were suffering from post-traumatic osteoarthritis, and the other two were suffering from patellofemoral pain (Table 1).

### 2.2. Eligibility Criteria

The study included patients with knee pain diagnosed with primary or secondary OA grades 2 to 4 based on the Kellgren and Lawrence scale [10], all of whom had previously received both corticoid and hyaluronic acid infiltrations. Patients were excluded if they had any autoimmune diseases, including rheumatoid arthritis, uncontrolled concomitant metabolic diseases, or the presence of a prior knee infection.

### 2.3. Rigenera^®^ Protocol

**Figure 1:**
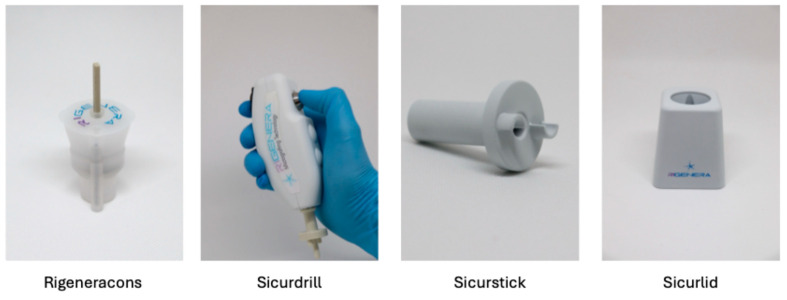
Rigenera^®^ devices used during the procedure.

Autologous micrografts were collected using the Class II-a CE medical device Rigeneracons (Human Brain Wave s.r.l, Turin, Italy) (Figure 1), while the rotation of Rigeneracons was triggered using Sicurdrill^®^ 2.0, Sicurlid, and Sicurstick (Figure 1).

**Figure 2:**
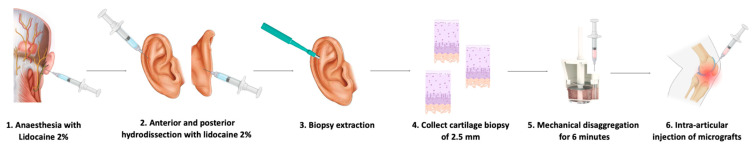
Rigenera^®^ technology protocol. The procedure begins with local anesthesia using 2% lidocaine (**1**), followed by the anterior and posterior hydro-dissection of the auricular cartilage (**2**). Cartilage biopsies (2.5 mm) are then extracted from the ear (**3**,**4**). The biopsies are mechanically disaggregated for 6 min (**5**) to obtain micrografts. Finally, the micrografts are injected intra-articularly into the target joint (**6**). Created with Biorender.com.

First, three biopsy samples were obtained from the retro-auricular zone using a 2.5 mm dermal punch. The area for biopsies was first cleaned with an antiseptic solution; 2.5 mL of Lidocaine 2% without vasoconstrictors was applied around the tissue to be biopsied. Biopsy samples were transferred into the medical device, and 4 mL of saline solution (0.9% Sodium Chloride injection B.P.) was added to the Rigeneracons. The samples were processed for 6 min with controlled rotations of 80 rpm. Afterward, the micrograft suspension was aspirated from the hole using syringes and injected into the area with a 5 mL Luer Lock Syringe with a needle of 21G 1/2, 0.8 × 40 mm [22]. The entire procedure was conducted under sterile conditions, and the Rigeneracons, intended for single use, were safely disposed of following local guidelines (Figure 2).

### 2.4. Assessment

X-rays were performed before the procedure. The Knee Injury and Osteoarthritis Outcome Score (KOOS) questionnaire [23], recommended by the International Knee Documentation Committee, was used to assess pain, stiffness, and function before the procedure and at 1, 6, and 12 months post-procedure. The KOOS is a 42-item self-reported measure that evaluates the patient’s perception of knee health, symptoms, and functionality. Each item offers five possible answers scored from 0 (no problems) to 4 (extreme problems), and scores are transformed to a 0–100 scale, where 0 indicates severe knee issues and 100 indicates no problems. Additionally, the Western Ontario and McMaster Universities Osteoarthritis Index (WOMAC) questionnaire [24] was administered before the procedure and at 1, 6, and 12 months afterward. This widely used index assesses pain, stiffness, and physical function related to daily activities such as walking, climbing stairs, and sitting down and standing up from chairs. WOMAC scores are also transformed to a 0–100 scale, where 0 represents no knee problems and 100 indicates significant knee pain or functional limitations. Furthermore, the Visual Analog Scale (VAS) [25,26] was used to evaluate pain intensity before the procedure and at 1, 6, and 12 months post-procedure. Patients rate their pain on a scale from 0 (no pain) to 10 (awful pain), providing a numeric score to quantify osteoarthritis-related pain severity. Throughout the study, adverse events were monitored. The biopsied area on the ear was regularly examined for signs of infection or abnormal healing.

### 2.5. Statistical Analysis

The count data were summarized as absolute numbers and proportions. Additionally, means and standard deviations were calculated for continuous variables. Data were collected and analyzed using Microsoft Excel for MAC (Microsoft, Washington, DC, USA).

## 3. Results

### 3.1. Pain Management

**Figure 3:**
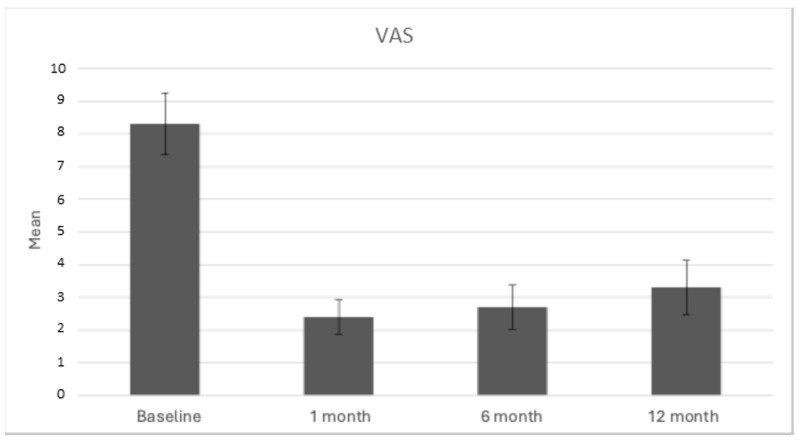
Mean VAS scores recorded at baseline and 1 month, 6 months, and 12 months post-treatment, illustrating the changes in pain perception over time.

The mean VAS score at baseline was 8.3 ± 0.94, indicating severe-to-awful pain among the participants. The distribution of VAS scores is shown in Figure 3. After 1 month post-procedure, the mean VAS score significantly decreased to 2.4 ± 0.51. This substantial reduction in pain was sustained over time, with mean VAS scores recorded at 2.7 ± 0.67 after 6 months and 3.3 ± 0.82 after 12 months, as illustrated in Figure 3.

### 3.2. Assessment of Joint Function and Symptoms: KOOS and WOMAC Scores

**Figure 4:**
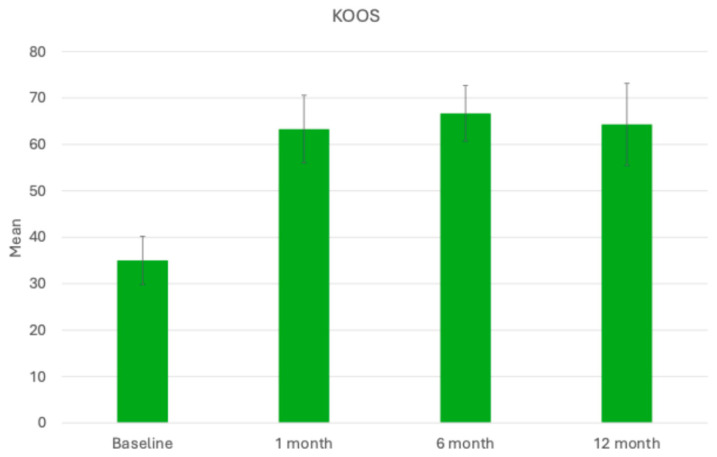
Mean KOOS scores recorded before the procedure, and at 1, 6, and 12 months post-procedure, highlighting changes in knee function and symptoms over the course of the follow-up period.

**Figure 5:**
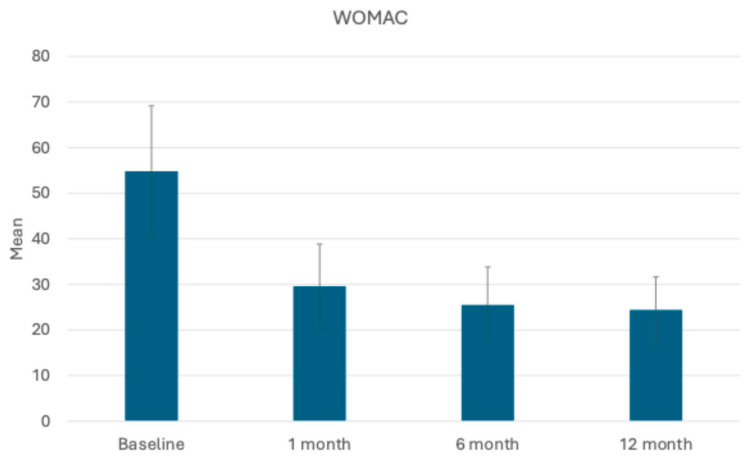
Mean WOMAC scores recorded before the procedure and at 1, 6, and 12 months post-procedure, demonstrating the improvements in knee function and symptoms during the follow-up period.

All 10 patients completed assessments at 1, 6, and 12 months post-operation. Improvements in the mean scores of both the KOOS total and WOMAC questionnaires revealed significant progress in managing pain and enhancing quality of life for the patients. The mean KOOS total score showed significant improvement over time, starting from 35 ± 5.18 at baseline and increasing to 63.3 ± 7.27 at 1 month, then to 66.7 ± 6 at 6 months, and then remaining stable even 12 months post-procedure (Figure 4). In parallel, the mean WOMAC total score demonstrated significant improvement over time, decreasing from 54.8 ± 14.36 at baseline to 29.6 ± 9.24 at 1 month. At the 6- and 12-month follow-ups, the mean WOMAC score decreased further with values of 25.5 ± 8.31 and 24.4 ± 7.2, respectively (Figure 5). These improvements reflect significant reductions in pain, stiffness, and physical dysfunction, indicating enhanced mobility and daily functioning for the patients. Overall, the WOMAC results underscore the effectiveness of the procedure in reducing symptoms and improving quality of life for patients over the 12-month follow-up period.

## 4. Discussion

In patients with OA, pain is a complex and multifaceted symptom that significantly affects quality of life [27,28]. This pain has multiple facets, including inflammatory and neuropathic characteristics [29]. It initially results from the degradation of the cartilage, which exposes the underlying bone and creates friction within the joint [30,31]. The disruption of the articular cartilage, which normally provides a smooth and lubricated surface for movement, causes a release of fragments and decomposition products from the matrix into the joint space, triggering an inflammatory response. The subsequent activation of cytokines sensitizes pain receptors and promotes the release of other pain-inducing substances [32,33]. This combination of factors makes OA pain management challenging, often requiring a comprehensive approach that includes medication, lifestyle changes, and surgical interventions [13,34]. Specifically, newly studied orthobiologic treatments may play a role in pain control associated with OA.

Some of these treatments utilize biological substances to modulate the inflammatory response and try to promote tissue repair. In this way, they try to modify the disease process and improve management of the symptoms, potentially offering long-term benefits for patients with OA [16,35]. Rigenera^®^ Technology is a procedure designed to harness the body’s regenerative capabilities to treat various conditions in the fields of dermatology, dentistry, orthopedics, and wound care. This technology focuses on the use of micrografts to reduce inflammation and promote tissue healing [36]. The role of micrografts has been explored in depth in previous in vitro and in vivo studies for the treatment of osteochondral defects [37,38], and the results of the present study indicate that treatment led to a significant reduction in pain in patients with knee osteoarthritis. In fact, the mean scores on the VAS decreased by 71% after 1 month post-procedure compared with the baseline, and these improvements were maintained for up to 12 months after the procedure. Similar results were obtained with KOOS scores, in which an increase of 80% was observed after the first month of follow-up compared to baseline, reaching a score of 83% one year after the procedure. In parallel, in the WOMAC scores, we also observed a reduction of 55% one year post-procedure. These data suggest that Rigenera^®^ Technology could represent an effective and safe therapeutic option for patients with knee osteoarthritis, especially in those who do not respond well to conventional treatments. In all patients, the ear wound was fully healed within 7 days of the procedure, with no adverse events reported either during the procedure or in the subsequent weeks and months. This approach could reduce the need for invasive surgery and improve the quality of life for patients.

This study presents some limitations, including the small number of patients treated, the short-term follow-up and the absence of a control group. However, due to the results shown, further studies with a statistically significant number of patients and two-year follow-up should be carried out to confirm the results presented.

## 5. Conclusions

This study indicates that treatment with autologous micrografts using Rigenera^®^ Technology is a safe and effective method for reducing pain and improving function in patients with knee osteoarthritis. These encouraging results highlight the need for further research to validate and expand the potential clinical applications of this therapeutic approach, promoting its broader use in clinical practice.

## Figures and Tables

**Table 1:** Baseline characteristics: demographics and disease profiles of the patients.

Patient Number	Age	Sex	BMI (kg/m^2^)	Diagnosis	Kellgren and Lawrence Scale Mean Score	Past Relevant Medications—Type and Treatment Period
1	64	F	31	Primary Osteoarthritis	3	Anti-Inflammatory Drugs, Corticoid Infiltration (1 time), HA Infiltration (2 times)
2	59	M	28	Post-Traumatic Osteoarthritis	4	Anti-Inflammatory Drugs, Corticoid Infiltration (3 times), HA Infiltration (3 times)
3	39	F	35	Patello-femoral Pain/Chondral Injuries	2	Anti-Inflammatory Drugs, Corticoid Infiltration (1 time), HA Infiltration (4 times)
4	66	F	25	Primary Osteoarthritis	3	Anti-Inflammatory Drugs, Corticoid Infiltration (5 times), HA Infiltration (4 times)
5	45	M	24	Post-Traumatic Osteoarthritis	3	Anti-Inflammatory Drugs, Corticoid Infiltration (3 times), HA Infiltration (3 times)
6	54	M	34	Primary Osteoarthritis	4	Anti-Inflammatory Drugs, Corticoid Infiltration (2 times), HA Infiltration (4 times)
7	64	F	32	Primary Osteoarthritis	3	Anti-Inflammatory Drugs, Corticoid Infiltration (1 time), HA Infiltration (1 time)
8	47	F	30	Patello-femoral Pain/Chondral Injuries	2	Anti-Inflammatory Drugs, Corticoid Infiltration (3 times), HA Infiltration (2 times), Genicular Nerve Block
9	55	M	28	Primary Osteoarthritis	3	Anti-Inflammatory Drugs, Corticoid Infiltration (3 times), HA Infiltration (2 times)
10	63	M	31	Primary Osteoarthritis	3	Anti-Inflammatory Drugs, Corticoid Infiltration (2 times), HA Infiltration (3 times), Genicular Nerve Block

## Data Availability

Data is contained within the article.
